# The role and therapeutic potential of mitophagy in major depressive disorder

**DOI:** 10.3389/fphar.2025.1564276

**Published:** 2025-03-26

**Authors:** Xin-Nuan Shi, Chen-Yue Liu, Lin Li, Ming-Li Yao, Zhen Zhong, You-Ming Jiang

**Affiliations:** ^1^ School of Life Sciences, Beijing University of Chinese Medicine, Beijing, China; ^2^ State Key Laboratory for Quality Ensurance and Sustainable Use of Dao-di Herbs, Institute of Chinese Materia Medica, China Academy of Chinese Medical Sciences, Beijing, China; ^3^ Traditional Chinese Medicine School, Beijing University of Chinese Medicine, Beijing, China

**Keywords:** major depressive disorder, mitophagy, mitophagy-related proteins, mitophagy-related pathways, therapeutic potential

## Abstract

Major depressive disorder, also known as MDD, affects more than 264 million people globally, making it a prevalent and critical health challenge. Traditional treatments show limited efficacy in many patients. Therefore, exploring new treatment methods is particularly crucial. Mitophagy, as a regulatory process, can help understand and treat MDD. This paper focuses on the molecular mechanisms of mitophagy, starting from proteins and related pathways, and its role in MDD. The study also explores the associations between mitophagy and neuroinflammation, oxidative stress, neurotransmitter synthesis, and neuroplasticity in MDD and discusses the progress of clinical research on the role of mitophagy in MDD. In addition, the article describes the current pharmaceutical and non-pharmaceutical interventions that can regulate mitophagy in MDD and unravels the potential and challenges of these therapeutic strategies in clinical settings. This article offers a deeper insight into the pathogenesis of MDD and offers a scientific basis for the development of new treatment strategies.

## 1 Introduction

Major depressive disorder (MDD) is a severe mood disorder characterized by persistent low mood, loss of interest or pleasure, and a range of physical and cognitive symptoms. Epidemiological studies have shown that the lifetime prevalence of MDD is 34%, making it a leading cause of the global disease burden (Kessler et al., 2012; Huang et al., 2019). It not only severely lowers the quality of life of patients but also imposes a significant socio-economic burden, increasing medical expenses, decreasing productivity, and incurring the costs associated with suicide (Whiteford et al., 2013). Despite the availability of various treatments, including pharmacotherapy and psychotherapy, a considerable proportion of patients do not respond to existing treatments, making the treatment of MDD challenging (Trivedi et al., 2006). Moreover, the high recurrence rate of MDD and poor long-term prognosis increases the complexity of treatment (Judd et al., 1998). Therefore, a deeper insight into the pathogenesis of MDD and the search for new therapeutic targets are crucial for improving treatment outcomes and the prognosis of patients.

Mitochondria are essential organelles in all eukaryotic cells, serving as the cellular energy power plant. They are the site for aerobic respiration and oxidative phosphorylation to produce Adenosine Triphosphate (ATP). Under physiological conditions, mitochondria function as energy factories, maintaining cellular functions and participating in physiological processes, such as cell growth and division, signal transduction, metabolic regulation, and apoptosis (Ma et al., 2020). Pathological conditions can damage mitochondria, which promotes mitophagy. Mitophagy protects cells against oxidative damage by removing damaged and dysfunctional mitochondria (Tan et al., 2016), thus contributing to cellular adaptation and survival (Ashrafi and Schwarz, 2013). Mitophagy can be categorized into basal mitophagy, stress-induced mitophagy, and programmed mitophagy. Stress conditions include hunger, hypoxia, etc. Stress-induced mitophagy is closely associated with the development and progression of various diseases, including neurodegenerative diseases, cardiovascular diseases, and mental disorders (Menzies et al., 2017; Zilocchi et al., 2020; Srivastava et al., 2018; Wang et al., 2021). Programmed mitophagy refers to the process of mitochondrial clearance that occurs according to a predetermined program during cell development, differentiation, or specific physiological processes. It plays a crucial role in maintaining cellular metabolic homeostasis and regulating cell fate (Srivastava et al., 2018). In this paper,Stress-induced mitophagy was reviewed.

This article aims to integrate and analyze existing research to explore the mechanisms of action and therapeutic potential of mitophagy in MDD. we will discuss the progress of clinical research on mitophagy in major depressive disorder, including its connections with neuroinflammation, oxidative stress, neurotransmitter synthesis, and neuroplasticity. In addition, we discuss the progress of clinical research on mitophagy in MDD and the potential therapeutic strategies for regulating mitophagy to treat MDD. By gaining a deeper understanding of the mechanisms underlying mitophagy in MDD, we hope to provide new insights and approaches for the treatment of this disorder.

## 2 Molecular mechanism of mitophagy

Mitochondria are continuously subjected to damage, and when these damaged mitochondria are not promptly removed, they can negatively affect cellular function and even lead to cell death. Mitophagy, as a self-protection mechanism, can identify and eliminate damaged or dysfunctional mitochondria, thereby maintaining the stability of the intracellular environment (Li et al., 2022). The inducing factors include the accumulation of reactive oxygen species (ROS) (Orekhov et al., 2023),reactive nitrogen species (RNS) (Caruso et al., 2019), inflammatory factors, such as Interleukin-6(IL-6), and Tumor Necrosis Factor-alpha (TNF-α), Interferon-gamma (IFN-γ), mitochondrial DNA mutations, nutrient deficiency (Dowlati et al., 2010), hunger, hypoxia, traumatic brain injury, viral infections, and metabolic disorders,etc (Menzies et al., 2017; Zilocchi et al., 2020; Wang et al., 2021). This process involves several steps: first, damaged mitochondria are recognized and tagged by specific receptor proteins. Subsequently, these mitochondria are sequestered within autophagosomes. Finally, the autophagosome fuses with the lysosome to form an autolysosome, where the mitochondria are degraded (Ashrafi and Schwarz, 2013). The regulatory mechanisms of mitophagy are complex, involving not only the direct participation of proteins but also the regulation of various signaling molecules, including the PTEN-Induced Kinase 1(PINK1)/Parkin RBR E3 Ubiquitin Protein Ligase (Parkin), Unc-51 Like Kinase 1(ULK1)/Autophagy-related gene 1(Atg1), Mammalian Target of Rapamycin (mTOR), and FUN14 Domain Containing 1(FUNDC1) signaling pathways, and the molecules associated with apoptosis (Li et al., 2023; Jung et al., 2010; Kim et al., 2011). These molecules play critical roles in response to changes in intracellular and extracellular environments, regulate signal transduction, and modulate the autophagy process, precisely regulating the initiation and progression of mitophagy.

### 2.1 Mitophagy-related proteins

The process of mitophagy is complex and regulated by several proteins. It can proceed through both ubiquitin-dependent and ubiquitin-independent pathways. PINK1 is one of the key regulatory genes in mitophagy in the ubiquitin-dependent pathway. The PINK1 protein encoded by the PINK1 gene accumulates on the membranes of damaged mitochondria, recruiting and activating the E3 ubiquitin ligase Parkin. Activation of PINK1 enhances the ubiquitination of mitochondrial proteins, marking the damaged mitochondria for autophagy (Lu et al., 2020; Choubey et al., 2021). Some studies have used C57BL/6 PINK1−/−mice, subjected to chronic restraint stress (CRS) and long-term corticosterone (CORT) treatment, and found that the absence of PINK1 significantly impacts adult hippocampal neurogenesis and striatal plasticity in mice, and leads to a decrease in dopamine release in the striatum (Liu et al., 2024a). Moreover, PINK1^−/−^ mice exhibited a lower threshold for depression-like behavior under chronic stress conditions, as evidenced by increased levels of behavioral despair in the forced swim test (FST) and tail suspension test (TST) (Liu et al., 2024b). These findings suggest that the loss of PINK1 not only impairs neuronal plasticity and neurotransmitter function in adult mice but also significantly increases the susceptibility to depression-like behaviors induced by chronic stress, indicating a close association between PINK1 deficiency and the development of depression.Parkin is another key gene associated with mitophagy, and Parkin is an E3 ubiquitin ligase. The activation of Parkin depends on the PINK1 signaling pathway. Once activated, Parkin promotes the ubiquitination of damaged mitochondria, thereby facilitating autophagosome formation. The expression and activity of Parkin directly affect the efficiency of mitophagy (Xiujuan and Yan, 2018). Studies have found that mutations in Parkin are associated with the incidence of Parkinson’s disease and MDD, indicating its importance in neuropsychiatric disorders (Imai and Lu, 2011; Zhang, 2024).

Additionally, nuclear dot protein 52 kDa, sequestosome 1, and optineurin are important proteins in the non-ubiquitination pathway. Jin X et al. found that mitophagy degradation mediated by NIX was impaired in the hippocampal neurons of Chronic Unpredictable Mild Stress (CUMS)-induced C57BL/6N mice, leading to the accumulation of damaged mitochondria. This increases the expression of Microtubule-Associated Protein 1 Light Chain 3 Beta II/I (LC3BII/I), and Translocase of Outer Mitochondrial Membrane 20 (TOM20) proteins (Jin et al., 2023). Studies have found that the expression of mitophagy-related proteins Parkin, Microtubule-Associated Protein 1 Light Chain 3 (LC3), and Sequestosome 1(P62) is upregulated in CUMS rats (Cheng et al., 2023). Lipopolysaccharide (LPS) and Chronic Social Defeat Stress (CSDS) downregulate the mRNA expression of BCL2/Adenovirus E1B 19 kDa-Interacting Protein 3-like (NIX) and Microtubule-Associated Protein 1 Light Chain 3 Alpha (LC3A) in the blood of C57BL/6N mice, while Optineurin and NBR1 Domain Containing 1(NDP52) proteins are not affected in CSDS (Lu et al., 2023).

The non-ubiquitin pathway primarily relies on mitophagy receptors, which are activated when ubiquitin chains accumulate to a certain level. These mitophagy adaptors have a ubiquitin-binding domain, which recognizes ubiquitin chains linked to the cargo, and an LC3-interacting region (LIR), which engages with the phagophore membrane wrapped with LC3B, thereby initiating mitophagy (Harper et al., 2018). The proteins include:

(1) BCL2/adenovirus E1B 19 kDa-interacting protein 3(BNIP3) is a BH3-only (Bcl-2 homology domain 3-only proteins) related to mitophagy, which enhances the recognition and degradation of damaged mitochondria by interacting with LC3 family proteins (Rikka et al., 2011). Mitophagy is excessively activated in the animal models of MDD, which affects the survival and function of neurons and significantly increases the expression level of BNIP3 (Tohda et al., 2010). NIX is a homolog of BNIP3 and plays an important role in mitophagy. NIX promotes mitophagy by interacting with LC3 (Zheng et al., 2017). Fang et al. found that the dysfunctional mitophagy receptor NIX may be a key molecular mechanism, by which TNF-α induces MDD (Lu et al., 2023), supporting the potential role of mitophagy in MDD.
(2) ULK1 is a serine/threonine kinase in mammals (Zhang et al., 2019). The ULK1 protein encoded by the ULK1 gene is activated in response to nutritional deficiency or stress, initiating autophagy by forming the ULK1-Atg13-FIP200 complex with Atg13 and FIP200 (Ganley et al., 2009). Studies have found that compared to normal rats, the phosphorylation level of ULK1 and the expression of Atg13 are upregulated in the hippocampus of depressed rats (Wang and Wang, 2023), suggesting that ULK1 plays a role in the pathogenesis of MDD.
(3) mTOR protein plays a pivotal role in the regulation of autophagy, and its function is affected by nutritional status and energy levels. mTOR activity decreases and ULK1 activity increases in depression, thereby inducing autophagy (Battaglioni et al., 2022; Dunlop and Tee, 2014). Decreased cellular energy levels activate AMP-activated protein kinase (AMPK), inhibit mTOR, and induce autophagy, thereby helping cells adapt to the energy-deficient environment (Ma et al., 2022). Additionally, calcium/calmodulin-dependent protein kinase kinase β (CaMKKβ) acts as a bridge in the regulation of autophagy, linking intracellular calcium signaling to energy sensing pathways (Green et al., 2011). Increased intracellular calcium ion concentration CaMKKβ activates AMPK, promoting the autophagy process.
(4) Silent information regulator 1 (SIRT1) is a deacetylase that affects the activity of various transcription factors through deacetylation, thereby directly inducing the expression of autophagy-related genes [such as Autophagy-related protein 5 (Atg5), Autophagy-related protein 7 (Atg7), and LC3] and promoting mitophagy (Ou et al., 2014).
(5) Translocated in Liposarcoma (FUS) is a multifunctional DNA/RNA-binding protein that supports the repair of DNA double-strand breaks (Jia et al., 2021) and regulates gene expression and cellular stress responses (Blechingberg et al., 2012). A proteomics-based study indicated that the expression level of the FUS gene is reduced in patients with MDD, affecting mitophagy. However, the study did not elaborate on its specific regulation of mitophagy (Blechingberg et al., 2012; Zhang et al., 2023). The baculoviral IAP repeat containing 2 (BIRC2) protein inhibits apoptosis and can promote the ubiquitination of Receptor-Interacting Serine/Threonine-Protein Kinase 1 (RIPK1), thereby inhibiting RIPK1-mediated apoptosis and inflammatory responses. Studies have shown that BIRC2 expression is upregulated in patients with MDD, thereby inhibiting mitophagy and affecting the health and function of neurons (Zhang et al., 2023).
(6) FUNDC1 is a mitophagy receptor that interacts with LC3B and promotes its recruitment to mitochondria during mitophagy (Liu et al., 2012). In normal physiological conditions, the phosphorylation of tyrosine 18 and serine 13 sites hinders FUNDC1-mediated mitosis. Hypoxia inactivates Src and leads to FUNDC1 dephosphorylation, leading to increased colocalization and interaction between FUNDC1 and LC3B. This results in the selective binding of mitochondria to the isolation membrane associated with LC3, thereby promoting the removal of mitochondria by Lysosome-associated Membrane Protein 1 (LAMP1)-positive autolysosomes (Chen et al., 2014; Lv et al., 2017).

### 2.2 Mitophagy-related pathways

The regulation of mitophagy is a complex process that relies not only on the expression of specific genes but also involves the coordinated function of several signaling pathways. These pathways regulate the initiation, progression, and termination of mitophagy through precise molecular mechanisms. Below are several key regulatory pathways:

(1) The PINK1/Parkin pathway: This is the most extensively studied pathway in mitophagy. The damaged mitochondria are first recognized by the PINK1/Parkin pathway. PINK1 accumulates on the outer membrane of the damaged mitochondria, subsequently recruiting and activating Parkin (Lazarou et al., 2015). The activation of Parkin promotes the ubiquitination of mitochondrial proteins, leading to the recruitment of autophagosomes. Subsequently, the autophagosome membrane extends and engulfs the damaged mitochondria, forming a closed autophagic vacuole. The autophagic vacuole fuses with the lysosome to form an autolysosome, in which the mitochondria are degraded and their components are recycled (Eiyama and Okamoto, 2015; Koyano et al., 2014).
(2) The ULK1-FIP200 complex pathway: ULK1 is a serine/threonine kinase. Nutritional deficiency or stress activates ULK1, which in turn activates Atg13 and FIP200, forming the ULK1-Atg13-FIP200 complex to initiate autophagy.
(3) The mTOR signaling pathway: The activity of mTOR is regulated by nutritional status and energy levels, and its inhibitory signals lead to the dephosphorylation of Atg13, thereby controlling the activity of ULK1 and the initiation of autophagy. Additionally, proteins encoded by the BNIP3 and NIX genes promote the recognition and degradation of damaged mitochondria by interacting with the LC3 family proteins (Trivedi et al., 2006). Beclin-1 interacts with the class III phosphatidylinositol 3-kinase (PI3K) complex to regulate autophagosome formation. Increased expression levels of Beclin-1 generally promote autophagy (McKnight and Yue, 2013). Small Guanine Nucleotide-binding Proteins (GTPases) of the Rab(Ras-related protein) family play a crucial role in the formation, maturation, and fusion of autophagosomes with lysosomes. Specific Rab proteins, such as Rab7 and Rab9, regulate the later stages of mitophagy to ensure the effective degradation of damaged mitochondria (Zhang et al., 2009).

## 3 The role of mitophagy in MDD

Mitophagy is involved in the development of various neurodegenerative diseases and mental disorders. Particularly, it not only alleviates neuroinflammation and oxidative stress and regulates neurotransmitter synthesis but also maintains neuroplasticity and the survival of neurons in MDD.

### 3.1 Mitophagy and neuroinflammation and oxidative stress

Mitophagy plays a positive role in alleviating neuroinflammation and oxidative stress in MDD. Damaged mitochondria are a major source of ROS and RNS. The accumulation of ROS and RNS leads to an inability of the antioxidant system to match the clearance capacity in a timely manner, resulting in exacerbated oxidative stress, which triggers cellular inflammation and tissue damage (Orekhov et al., 2023; Wen et al., 2025). This leads to a significant increase in the levels of malondialdehyde in the plasma and a decrease in the activity of antioxidant enzymes such as superoxide dismutase (SOD). The reaction of nitric oxide (NO) with superoxide anions generates peroxynitrite, a potent oxidizing agent that damages biomacromolecules and further exacerbates cellular dysfunction (Caruso et al., 2019). Mitophagy can maintain cellular redox balance and reduce the release of inflammatory mediators by clearing these damaged mitochondria, thereby significantly influencing the development of MDD (Liu et al., 2024b). The mechanism may be related to the inhibition of Nuclear Factor kappa-light-chain-enhancer of activated B cells (NF-κB) signaling pathway (Zhong et al., 2016). Additionally, mitophagy can reduce the opening of the mitochondrial permeability transition pore (mPTP), thereby alleviating oxidative stress and cell death (Karch et al., 2015).

### 3.2 Mitophagy and neurotransmitter synthesis

In MDD, the effect of mitophagy on neurotransmitter synthesis can be reflected in the following aspects: (1) Mitophagy can ameliorate the insufficiency of the cellular respiratory chain, enhance ATP production after mitochondrial dysfunction, and reduce energy supply for neurotransmitter synthesis (Ming-Xi and De-Zhi, 2017). For example, downregulation of neurotransmitters, such as dopamine, 5-Hydroxytryptamine(5-HT) (serotonin), and norepinephrine, which are associated with mood regulation and cognitive function, was found to be associated with mitochondrial dysfunction in patients with MDD (Yao et al., 2023; Graves et al., 2020; Tian et al., 2023). (2) Mitophagy can manifest by altering the sensitivity and signal transduction of neurotransmitter receptors. The downregulation or desensitization of neurotransmitter receptors may weaken the effects of neurotransmitters in MDD (Carr and Lucki, 2011). Mitophagy maintains the normal function and signal transduction efficiency of neurotransmitter receptors by preserving the quality and quantity of intracellular mitochondria. Some studies have reported that the early incidence of rapid eye movement (REM) sleep in patients with MDD may be due to the hyperactivity of the cholinergic system. This change in sleep pattern may affect the sleep pattern of patients with MDD by regulating the neurotransmitter system (Zhu et al., 2024; Riemann et al., 2020). These findings suggest that the symptoms of MDD may be associated with fluctuations in neurotransmitter levels. (3) The effect of mitophagy on neurotransmitter synthesis also involves the adaptive regulation of the neurotransmitter system. In prolonged depression, the neurotransmitter system must undergo adaptive changes to maintain emotional stability (Weiwei and Yu, 2019). A study explored the relationship between changes in brain-derived neurotrophic factor (BDNF) levels and cognitive decline among elderly patients with depression and mild cognitive impairment. The study found that compared to cognitively normal elderly individuals who never had depression, BDNF levels significantly decreased over time in elderly patients with depression. This suggests that reduced BDNF levels may be associated with cognitive decline (Diniz et al., 2014). This suggests that upregulating BDNF levels by activating mitophagy may improve these cognitive symptoms.

From the perspective of disease development, the regulatory role of mitophagy in MDD profoundly affects the long-term prognosis of the disease. Persistent mitochondrial dysfunction and neurotransmitter imbalance can exacerbate neuronal damage and lead to continuous cognitive decline, while effective mitophagy can prevent these neurodegenerative changes (Lou et al., 2020). Furthermore, the activation of mitophagy can prevent the relapse and chronicity of MDD, as it helps maintain the stability and adaptability of the neurotransmitter system (Xu et al., 2023).

### 3.3 Mitophagy, neuroplasticity, and neuronal survival

In a healthy state, mitophagy supports neuroplasticity and neuronal survival by clearing damaged mitochondria, reducing ROS generation, and maintaining mitochondrial function and the homeostasis of the intracellular environment (Sayehmiri et al., 2024). Neuroplasticity is impaired in depression, leading to cognitive dysfunction and abnormal emotional regulation (Rădulescu et al., 2021). Mitophagy maintains the integrity of synaptic structure and function by removing damaged mitochondria, thereby positively affecting neuroplasticity (Han et al., 2020).

Depression damages neurons due to insufficient energy supply, increased oxidative stress, and activation of cell death signaling, thereby affecting emotional regulation and cognitive function (Yang et al., 2024). Mitophagy protects neurons against damage by removing damaged mitochondria, reducing oxidative stress, and inhibiting cell death signaling (Li et al., 2021).

Additionally, mitophagy can be regulated by neurotrophic factors, such as BDNF. BDNF is a secretory protein that can affect neuronal survival, synaptic plasticity, and the generation of new neurons (Geng et al., 2023). Upregulation of BDNF can enhance the adaptability of the neurotransmitter system, thereby increasing the resistance of neurons to stress. The expression of BDNF decreases in depression (Jiake and Bai, 2014), while activation of mitophagy can improve neuroplasticity and neuronal survival by increasing the expression of BDNF (Zhang et al., 2021). Furthermore, mitophagy may also regulate neuroplasticity and neuronal survival through other neurotrophic factors, such as nerve growth factor (NGF) (Goglia et al., 2024; Chen et al., 2015). These neurotrophic factors play a crucial role in regulating neuron survival, differentiation, and synaptic function, and mitophagy can affect neuroplasticity and the survival of neurons by modulating the expression and function of these factors. The role of mitochondrial autophagy in MDD is shown in (Table 1).

**TABLE 1:** The role of mitophagy in MDD.

The pathways linking mitophagy to MDD	Mechanism of action	References
Neuroinflammation	Reduced release of inflammatory mediators and inhibition of the NF-κB signaling pathway	Zhong et al. (2016)
Oxidative stress	Removal of ROS, maintenance of intracellular redox balance, and decrease of the opening of the mitochondrial permeability transition pore (mPTP)	Caruso et al. (2019), Karch et al. (2015)
Neurotransmitter synthesis	Increased ATP production and increased energy supply for neurotransmitter synthesis	Ming-Xi and De-Zhi (2017)
	Increased sensitivity and signal transduction of neurotransmitter receptors	Carr and Lucki (2011)
Neuroplasticity	Reduced ROS levels, maintenance of mitochondrial function and intracellular homeostasis, and supporting neuroplasticity	Sayehmiri et al. (2024), Han et al. (2020)
Neuron survival	Removal of damaged mitochondria, suppression of oxidative stress, and inhibition of cell death signaling, thereby protecting neurons against damage	Li et al. (2021)
	Promoting BDNF expression	Jiake and Bai (2014), Zhang et al. (2021)
	Promoting NGF expression	Goglia et al. (2024), Chen et al. (2015)

### 3.4 Advances in clinical research on mitophagy and MDD

Recently, clinical research on the role of mitophagy in MDD has gained much attention; however, due to the lack of techniques for directly assessing mitophagy in clinical settings, researchers often rely on peripheral blood markers or indirect indicators, such as inflammatory factors, to assess the status of mitophagy. Scholars have found that the expression levels of mitophagy-related genes PINK1 and Parkin in the peripheral blood mononuclear cells (PBMCs) are significantly downregulated and negatively correlated with disease severity in patients with MDD (Scaini et al., 2022). Patients with MDD had significantly higher baseline levels of the mitochondrial autophagy-related protein Beclin-1 in peripheral blood compared to treatment responders, and these levels were significantly negatively correlated with treatment response (He et al., 2019). Another study found that the expression of SIRT1 in the peripheral blood of patients with MDD was significantly downregulated by 37% compared to the control group (Luo and Zhang, 2016). Through bioinformatics methods and machine learning algorithms, Zhang et al. identified mitophagy genes associated with MDD, namely, Matrin 3 (MATR3), Actin-like 6A (ACTL6A), FUS, BIRC2, and RIPK1 (Zhang et al., 2023). Notably, the study by Munshi et al. (2024) further elucidated the role of mitochondrial dynamics in MDD, particularly focusing on the key proteins involved in mitochondrial fusion and fission, Mitofusin-2 (MFN2) and Dynamin-related protein 1 (DNM1L). Mitochondrial fusion and fission are essential processes for maintaining mitochondrial morphology, function, and quantity, with MFN2 and DNM1L playing crucial roles in these processes. MFN2 primarily promotes mitochondrial fusion, helping to form a more efficient energy-producing network when there is a high energy demand. In contrast, DNM1L is involved in mitochondrial fission, aiding in the isolation and removal of damaged mitochondria to maintain a healthy mitochondrial state within the cell. The study found that (Munshi et al., 2024), compared to healthy controls, MDD patients exhibited a significant increase in MFN2 mRNA expression levels in Peripheral Blood Mononuclear Cells, while DNM1L expression levels showed no significant differences. This suggests that mitochondrial fusion is enhanced in MDD patients, while mitochondrial fission remains relatively stable. The increase in MFN2 reflects the cell’s adaptive response to energy demands and may also be related to the activation of mitophagy, thus highlighting MFN2’s important role in regulating mitophagy.

In summary, mitophagy is closely associated with MDD. In fact, activation of mitophagy can improve the treatment of MDD, providing patients with new options beyond traditional antidepressants. Clinical research progress on mitophagy and MDD is shown in (Table 2).

**TABLE 2:** Clinical research progress on mitophagy and MDD.

Disease	Total number of cases	Source of samples	Mitophagy indicators	Research methods	Medications used	References
MDD	77 cases	Peripheral blood mononuclear cells	Reduction in PINK1 and Parkin			Scaini et al. (2022)
MDD	186 cases	Peripheral blood mononuclear cells	Upregulation of mitochondrial dynamics-regulating gene MFN2, no significant differences in mitochondrial dynamics-regulating gene DNM1L			Munshi et al. (2024)
MDD	40 cases	Serum sample	Upregulation of Beclin-1	ELISA	Selective serotonin reuptake inhibitors (SSRIs) or serotonin-norepinephrine reuptake inhibitors (SNRIs)	He et al. (2019)
MDD	144 cases		Upregulation of MATR3, ACTL6A, FUS, BIRC2, and RIPK1	Consensus clustering analysis, differential expression gene (DEG) identification, functional enrichment analysis, co-expression network analysis, protein-protein interaction (PPI) network analysis		Zhang et al. (2023)
MDD	100 cases	Peripheral blood mononuclear cells	Downregulation of SIRT1	qPCR, WB		Luo and Zhang (2016)

## 4 Therapeutic potential of mitophagy

Mitophagy, as an active research field, has shown its therapeutic value in the treatment of MDD. Recently, an increasing number of studies have focused on developing drugs that can regulate mitophagy to improve the symptoms of MDD. The following sections will detail several major drugs and their mechanisms of action.

### 4.1 Antioxidants

Antioxidants play an important role in regulating mitophagy. Mitophagy suppresses oxidative stress by removing damaged mitochondria. N-acetylcysteine (NAC) can inhibit mitophagy by regulating GSK-3β/Drp1-mediated mitochondrial fission and inhibiting the expression of beclin-1 and the conversion of LC3 (Ali et al., 2022). The drug NAC not only showed satisfactory effects *in vitro* experiments but also exhibited significant antidepressant effects in animal models (Yang and Jie, 2016; Chakraborty et al., 2020). Additionally, coenzyme Q10 (CoQ10) can promote mitophagy by upregulating the levels of autophagy-related proteins (Atg5, beclin-1, LC-3II/LC3-I ratio), thereby reducing mitochondrial damage and cell death, and exerting neuroprotective effects (Liang et al., 2017). Studies have shown that CoQ10 exhibits certain therapeutic effects in patients with MDD (Majmasanaye et al., 2024).

### 4.2 Autophagy inducers

Autophagy inducers are a class of drugs that can directly activate mitophagy, among which, rapamycin has been extensively studied. Some studies provided preliminary evidence for the application of mitophagy in the treatment of MDD. Case reports indicated that rapamycin, as an mTOR pathway inhibitor, is a classic autophagy inducer and simultaneously enhances mitophagy by increasing the translocation of p62 and Parkin to damaged mitochondria. It has been found that rapamycin and its analogs can exert antidepressant effects by enhancing mitophagy to remove damaged mitochondria and restore mitochondrial function (Liu et al., 2024a). These findings support the concept that mitophagy activators may effectively treat patients with treatment-resistant depression. Additionally, patients with MDD receiving rapamycin may experience improvements in mood and cognitive function. Small animal experiments (Zheng et al., 2024) utilized C57BL/6J mice to establish a CRS model. CRS was successfully induced by subjecting the mice to 2 h of restraint stress daily for 14 consecutive days. The results showed that long-term intraperitoneal injection of rapamycin (1.0 mg/kg) significantly prevented depressive-like behaviors in CRS mice, as evidenced by the forced swimming test and sucrose preference test. Additionally, rapamycin increased myelination in the prefrontal cortex of CRS mice, as indicated by significant increases in myelin basic protein (MBP) and 2′,3′-cyclic nucleotide 3′-phosphodiesterase (CNP) levels. In another study (Zhang et al., 2024), Zhang et al. found that Alpha-lipoic acid (ALA) (5 mg/kg, once a day) administered to 2-month-old male APP23/PS45 mice for 4 months significantly improved the cognitive deficits of APP23/PS45 mice and activated BNIP3L-mediated mitochondrial autophagy.

Furthermore, other autophagy inducers, such as lithium salts, can exert neuroprotective effects and enhance neuroplasticity by regulating mitophagy, suggesting their potential as targets for treating depression. ALA can also improve depressive symptoms by increasing the expression of A Disintegrin and Metalloprotease 10 (ADAM10) α-secretase through mitophagy (Zhang et al., 2024). Metformin can induce mitophagy by upregulating the formation of PINK1/acidic vesicles and autophagosomes (Bhansali et al., 2020). Studies have found that metformin-mediated increase in mitophagy can improve the cognitive abilities of patients with MDD and diabetes (Guo et al., 2014). In addition, natural compounds, such as apigenin, can increase the expression of autophagy-related proteins, including Beclin1, ATG7, and LC3II, thereby promoting mitophagy (Hsu et al., 2021). These interventions reveal potential pathways for treating MDD by regulating mitophagy. However, due to the small sample sizes of these studies and the lack of rigorous validation from randomized controlled trials (RCTs), further studies are needed to confirm these preliminary findings.

### 4.3 Traditional antidepressants

Traditional antidepressants have also been found to exert antidepressant effects by regulating mitophagy. Selective Serotonin Reuptake Inhibitors (SSRIs), such as fluoxetine, can induce autophagy in microglia and increase the expression of autophagy-related proteins ATG5, LC3-II, and BDNF (Zhang et al., 2023). Furthermore, fluoxetine was shown to eliminate damaged mitochondria by promoting autophagy, reducing cell death, and finally improving pathological changes in hippocampal astrocytes (Shu et al., 2019). Animal experiments have indicated that treating depression with the tricyclic antidepressant imipramine can increase the mRNA levels of BNIP3 in the prefrontal cortex of learned helpless ICR Mouse (Tohda et al., 2010). These studies all suggest that traditional antidepressants promote mitophagy in the brain. The aforementioned drugs targeting mitophagy have shown positive effects in cell culture and animal models. Future studies should optimize drug dosages and explore new strategies for combination therapy. Furthermore, the development of individualized treatment plans is a major direction for future research. Through the application of genetic testing and biomarkers, the most suitable treatment plan can be tailored for each patient.

### 4.4 Non-pharmacological treatments

There are few studies on the correlation between non-pharmacological interventions for MDD and mitophagy. Studies have shown that regular physical exercise can significantly enhance mitochondrial function, upregulate mitophagy (Review on Exercise and Mitochondrial Dynamics, 2016; Laker et al., 2017), and alleviate depressive symptoms (Ross et al., 2023). Moreover, exercise can upregulate mitophagy, reduce the accumulation of damaged mitochondria by activating the Fibronectin Type III Domain Containing 5(FNDC5)/Irisin-PINK1/Parkin-LC3Ⅱ/L-P62 signaling pathway (Wu et al., 2023), and increase the expression of SIRT3 (Cheng et al., 2016), thereby improving depression. Additionally, caloric restriction (Mehrabani et al., 2020) and intermittent fasting (Kim and Lemasters, 2011) have been proven to stimulate mitochondrial biogenesis and improve mitophagy. Although exercise, caloric restriction, and intermittent fasting were shown to effectively enhance mitophagy and alleviate depressive symptoms, previous research findings have only touched the tip of the iceberg. The specific mechanisms, by which non-pharmacological interventions improve MDD through mitophagy need further in-depth research. Overall, the relationship between non-pharmacological interventions for MDD and mitophagy is a field with research value. More discoveries can be anticipated on how non-pharmacological interventions treat MDD by improving mitophagy, which will provide more treatment options for patients with MDD.

### 4.5 Gene-targeted drugs

Gene-targeted drugs have become a new therapeutic option. Scientists are increasingly focusing on the regulation of mitophagy-related genes, particularly genes linked to the PINK1/Parkin pathway. The PINK1 protein encoded by the PINK1 gene accumulates on the mitochondrial membrane and activates Parkin after mitochondrial damage. This mechanism lays the foundation for the clearance of damaged mitochondria. To maintain the stability of the intracellular environment, it is extremely important to promptly clear damaged mitochondria, especially in patients with MDD. Therefore, enhancing the activity of this pathway through drugs or gene editing techniques can help remove damaged mitochondria, thereby improving mitochondrial dysfunction associated with MDD. BNIP3 and NIX are two other key genes in the process of mitophagy, with equal importance in autophagy. Proteins encoded by the BNIP3 and NIX genes facilitate the recognition and degradation of damaged mitochondria by interacting with the LC3 family proteins. This mechanism can help maintain mitochondrial quality control; therefore, upregulating the expression of BNIP3 and NIX genes may promote the removal of damaged mitochondria and restore the balance of the intracellular microenvironment. Furthermore, the roles of the MATR3, ACTL6A, FUS, BIRC2, and RIPK1 genes, as potential biomarkers of MDD, in mitophagy needs necessitates future studies (Zhang et al., 2023). The association between mitophagy-related genes and MDD provides a new perspective for the diagnosis and treatment of MDD, aiding in early diagnosis and evaluation of treatment efficacy.

### 4.6 Future prospects for the development of drugs targeting mitophagy in MDD

**FIGURE 1:**
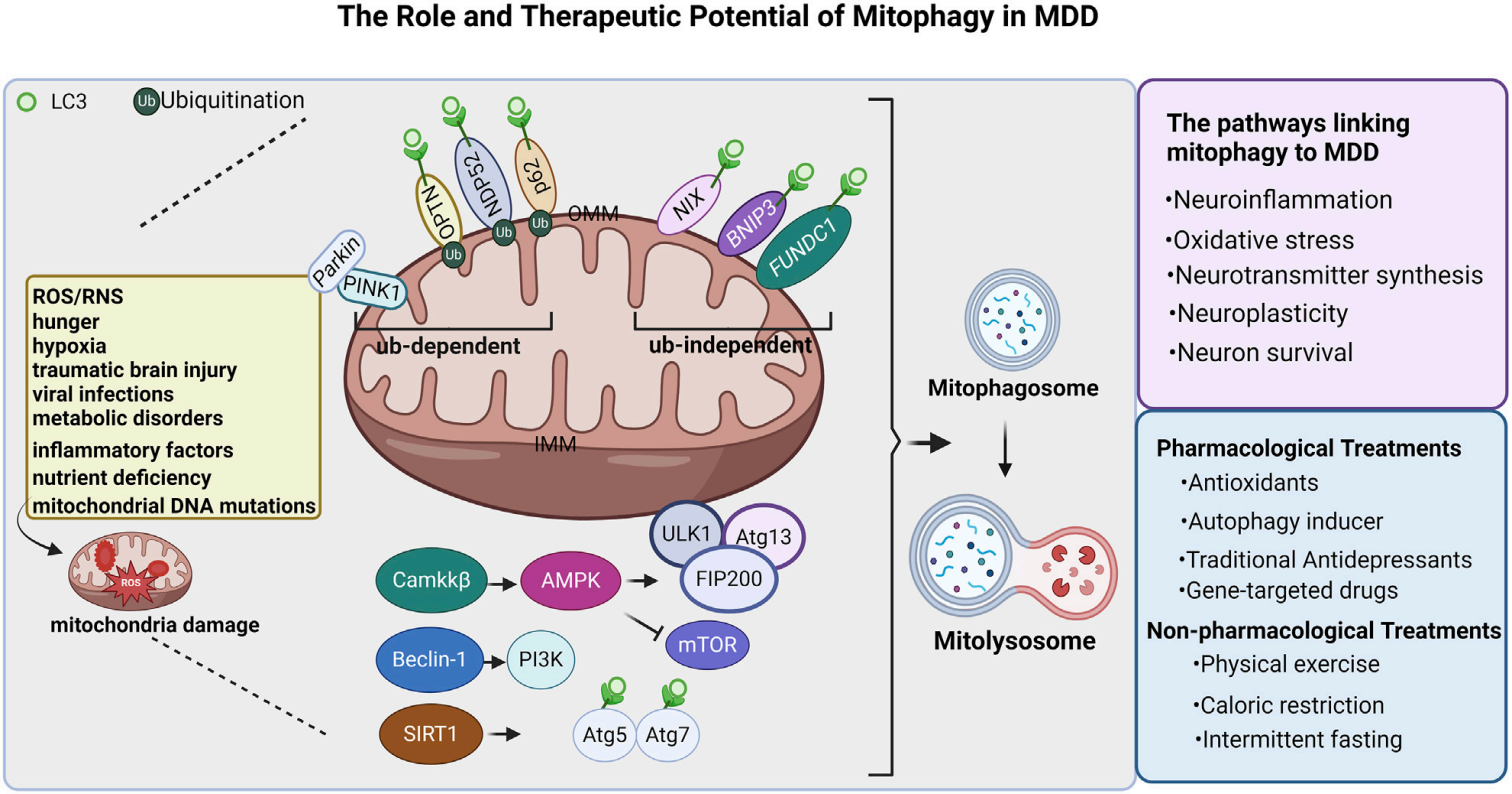
Regulatory mechanism of mitochondrial autophagy in major depressive depression. Mitophagy is essential for maintaining mitochondrial physiological function. In the case of mitochondrial damage caused by the accumulation of ROS, RNS,inflammatory factors, mitochondrial DNA mutations, and nutrient deficiency, hunger, hypoxia, traumatic brain injury, viral infections, and metabolic disorders. Defective mitochondrial clearance depends on ubiquitination and non-ubiquitination pathways and is engulfed by autophagosomes, called mitophagosomes. The Mitophagosome fuses with the lysosome, and the lysosomal enzyme degrades the mitochondria to form the Mitolysosome. In the ubiquitination (Ub)-dependent pathway, PINK1/Parkin, NDP52, P62, and OPTN initiate autophagy by binding to LC3; In the ubiquitination-independent pathway, NIX, BNIP3, and FUNDC1 initiate mitophagy by binding to LC3. In addition, Camkkβ promotes AMPK expression, promotes the expression of the complex ULK1, Atg13, and FIP200 to promote mitochondrial autophagy; Beclin1 promotes PI3K expression, and SIRT1 promotes Atg5 and Atg7 to bind to LC3 to promote mitochondrial autophagy. Mitochondrial autophagy is involved in MDD, Neuroinflammation, Oxidative stress, Neurotransmitter synthesis, Neuroplasticity, Neuron survival. Currently, there are pharmacological and nonpharmacological treatments that can modulate mitophagy. ROS: reactive oxygen species; OMM: Outer mitochondrial membrane; IMM: Inner mitochondrial membrane; Ub: ubiquitination. The figure was created with Biorender.com.

Future studies should focus on developing specific drugs targeting proteins associated with mitophagy, signaling molecules, and anti-inflammatory agents. Although these potential therapeutic strategies are theoretically appealing, they still need rigorous preclinical and clinical studies to validate their safety and efficacy. Only through these studies, we can develop new treatments for MDD. The figure illustrating the mechanisms of the role and therapeutic potential of mitophagy in MDD is shown in (Figure 1).

## 5 Prospects and challenges of clinical applications

Targets of mitophagy, such as Beclin-1, MATR3, ACTL6A, FUS, BIRC2, RIPK1, Parkin, LC3-II, SIRT1, etc., have been studied as biomarkers in clinical research (Table 3). Alongside scales, such as the Hamilton Depression Scale (HAMD) and Self-Rating Depression Scale (SDS), these biomarkers can serve as important evaluation indicators. They play a crucial role in the early detection, diagnosis, and objective evaluation of MDD.

**TABLE 3:** Mitophagy as a therapeutic target for MDD.

Category	Name	Mechanism of action	References
Antioxidants	N-acetylcysteine	Regulating GSK-3β/Drp1-mediated mitochondrial fission, inhibiting the expression of beclin-1 and the conversion of LC3, and suppressing mitophagy	Ali et al. (2022)
	Coenzyme Q10 (CoQ10)	Upregulating the expression levels of autophagy-related proteins (Atg5, beclin-1, and LC-3II/LC3-I ratio) to promote mitophagy	Liang et al. (2017)
Autophagy inducer	Rapamycin	Inhibiting the mTOR pathway, increasing the translocation of p62 and Parkin to damaged mitochondria to enhance mitophagy	Liu et al. (2024b)
	Metformin	Upregulating the formation of PINK1/acidic vesicles and autophagosomes to induce mitophagy	Bhansali et al. (2020)
	Apigenin	Increasing the expression of autophagy-related proteins, including Beclin1, ATG7, and LC3II, and promoting mitophagy	Hsu et al. (2021)
Traditional Antidepressants	Fluoxetine	Inducing autophagy and increasing the expression of autophagy-related proteins ATG5, LC3-II, and BDNF	Zhang et al. (2023)
	Imipramine	Promoting BNIP3 expression	Tohda et al. (2010)
Non-pharmacological Treatments	Physical exercise	Activating the FNDC5/Irisin-PINK1/Parkin-LC3Ⅱ/L-P62 signaling pathway and upregulating mitophagy	Cheng et al. (2016)
	Caloric restriction	Stimulating mitochondrial biogenesis and enhancing mitophagy	Mehrabani et al. (2020)
	Intermittent fasting	Stimulating mitochondrial biogenesis and enhancing mitophagy	Kim and Lemasters (2011)
Gene-targeted drugs	Drugs targeting the PINK1 gene and regulating BNIP3, NIX, MATR3, ACTL6A, FUS, BIRC2, and RIPK1	Promoting mitophagy	Lu et al. (2020), Choubey et al. (2021), Zhang et al. (2023)

The prospects of mitophagy modulation strategies in the treatment of MDD are mainly reflected in the following aspects: firstly, the realization of precision medicine. With the rapid development of genomics and bioinformatics, researchers can more accurately identify gene variants associated with mitophagy in MDD. This not only helps early diagnosis but also provides personalized treatment plans for patients. Targeting specific mitophagy pathways can effectively improve the symptoms of patients. Secondly, breakthroughs in drug development: Many studies have recently focused on developing drugs that can regulate mitophagy. These drugs may enhance mitophagy and remove damaged mitochondria, thereby improving neural function and alleviating depressive symptoms (Ghosh and Kumar, 2024). Despite the progress, the development of drugs targeting mitophagy will expand therapeutic options for MDD. With in-depth research into the mechanisms of mitophagy, more innovative drugs are expected to develop, more precisely targeting mitochondrial dysfunction and more effectively treating MDD. Secondly, mitophagy regulation can be used in conjunction with existing antidepressants to enhance the therapeutic effects. For example, combining pharmacological treatment with lifestyle interventions (such as exercise, dietary adjustments, etc.) can more effectively improve the overall health of patients. Finally, by monitoring the changes in mitophagy-related biomarkers, early intervention can be adopted before MDD symptoms appear. This preventive strategy can delay or even prevent disease progression, which is especially critical for high-risk groups, such as patients with a family history of MDD.

Although mitophagy regulatory strategies showed great promise in the treatment of MDD, there are still many challenges in their clinical application. The complexity of mechanistic studies increases the difficulty and uncertainty of drug development. Clinical trials also have their own limitations. There are few large-scale, multicenter randomized controlled studies. In addition, individual differences, such as patients’ genetic backgrounds, metabolic states, and lifestyles, lead to variations in the efficacy of mitophagy regulatory strategies. The lack of long-term safety assessments does not allow for a thorough evaluation of the impact on patients’ overall health and quality of life. Moreover, the lack of interdisciplinary collaboration in fields, such as neuroscience, genetics, and pharmacology has limited research on mitophagy.

In summary, strategies for regulating mitophagy have proven effective in the treatment of MDD, but they also face numerous challenges. Future clinical trials based on the experimental findings and more robust interdisciplinary collaboration are needed to unleash the full potential of mitophagy regulators in the management of MDD.

## 6 Conclusion

MDD is a chronic condition affecting millions of people worldwide. Despite some progress in recent years, current treatment options remain limited and do not sufficiently alleviate the symptoms of many patients with depression. Therefore, there is an urgent need for innovative therapeutic strategies. Based on the current advancements, restoring mitophagy levels may be an innovative approach to enhance the treatment efficacy for MDD.

Mitophagy regulates several biological processes, including neuroinflammation, oxidative stress, neurotransmitter synthesis, and neuroplasticity, involving various pathways and numerous genes and proteins. Changes in these genes, proteins, and pathways can directly affect the survival and function of neurons. Based on these mitophagy-related proteins and pathways, various drugs and intervention strategies have been developed, including antioxidants, autophagy inducers, and traditional antidepressants. In addition, non-pharmacological treatments, such as exercise, caloric restriction, and intermittent fasting, were shown to modulate mitophagy in MDD. Clinical studies have also shown that new strategies that regulate mitophagy can effectively and safely improve the symptoms of patients with MDD. In summary, mitophagy plays a key role in the pathogenesis of MDD and has become a crucial target for the treatment of MDD, bringing new hope to patients with MDD.

## Glossary

5-HT: 5-Hydroxytryptamine
ACTL6A: Actin-like 6A
ADAM10: A Disintegrin and Metalloprotease 10
ALA: Alpha-lipoic Acid
AMPK: AMP-activated protein kinase
ATP: Adenosine Triphosphate
Atg1: Autophagy-related gene 1
Atg13: Autophagy-related protein 13
Atg5: Autophagy-related protein 5
Atg7: Autophagy-related protein 7
BDNF: Brain-Derived Neurotrophic Factor
BH3-only: Bcl-2 homology domain 3-only proteins
BNIP3: BCL2/adenovirus E1B 19 kDa-interacting protein 3
BIRC2: Baculoviral IAP repeat-containing 2
CaMKKβ: Calcium/calmodulin-dependent protein kinase kinase β
CNP: 2′,3′-Cyclic Nucleotide 3′-Phosphodiesterase
CORT: Corticosterone
CRS: Chronic Restraint Stress
CSDS: Chronic Social Defeat Stress
CUMS: Chronic Unpredictable Mild Stress
CoQ10: Coenzyme Q10
DNM1L: Dynamin-related protein 1
FIP200: Focal Adhesion Kinase Family Interacting Protein of 200 kDa
FNDC5: Fibronectin Type III Domain Containing 5
FST: Forced Swim Test
FUNDC1: FUN14 Domain Containing 1
FUS: Translocated in Liposarcoma
GTPases: Guanine Nucleotide-binding Proteins
HAMD: Hamilton Depression Scale
IL-6: Interleukin-6
IFN-γ: Interferon-gamma
IMM: Inner Mitochondrial Membrane
LC3: Microtubule-Associated Protein 1 Light Chain 3
LC3A: Microtubule-Associated Protein 1 Light Chain 3 Alpha
LC3BII/I: Microtubule-Associated Protein 1 Light Chain 3 Beta II/I
LAMP1: Lysosome-associated Membrane Protein 1
LIR: LC3-interacting region
LPS: Lipopolysaccharide
MA: 3-Methyladenine
MATR3: Matrin 3
MDD: Major Depressive Disorder
MBP: Myelin Basic Protein
MFN2: Mitofusin-2
mPTP: Mitochondrial Permeability Transition Pores
mTOR: Mammalian Target of Rapamycin
NAC: N-acetylcysteine
NDP52: NBR1 Domain Containing 1
NF-κB: Nuclear Factor kappa-light-chain-enhancer of activated B cells
NGF: Nerve Growth Factor
NIX: BCL2/Adenovirus E1B 19 kDa-Interacting Protein 3-like
NO: Nitric Oxide
OMM: Outer Mitochondrial Membrane
PARKIN: Parkin E3 Ubiquitin Protein Ligase
PBMCs: Peripheral Blood Mononuclear Cells
PINK1: PTEN-Induced Kinase 1
PI3K: Phosphatidylinositol 3-Kinase
P62: Sequestosome 1
RAB: Ras-related protein
RCTs: Randomized Controlled Trials
REM: Rapid Eye Movement
RIPK1: Receptor-Interacting Serine/Threonine-Protein Kinase 1
RNS: Reactive Nitrogen Species
ROS: Reactive Oxygen Species
SDS: Self-Rating Depression Scale
SIRT1: Silent information regulator 1
SOD: Superoxide Dismutase
SSRIs: Selective Serotonin Reuptake Inhibitors
TST: Tail Suspension Test
TNF-α: Tumor Necrosis Factor-alpha
TOM20: Translocase of Outer Mitochondrial Membrane 20
Ub: Ubiquitination
ULK1: Unc-51 Like Kinase 1
